# Kappa-Carrageenan/Chitosan/Gelatin Scaffolds Provide a Biomimetic Microenvironment for Dentin-Pulp Regeneration

**DOI:** 10.3390/ijms24076465

**Published:** 2023-03-30

**Authors:** Konstantinos Loukelis, Foteini Machla, Athina Bakopoulou, Maria Chatzinikolaidou

**Affiliations:** 1 Department of Materials Science and Technology, University of Crete, 70013 Heraklion, Greece; 2 Department of Prosthodontics, School of Dentistry, Faculty of Health Sciences, Aristotle University of Thessaloniki, 54124 Thessaloniki, Greece; 3 Foundation for Research and Technology Hellas-Institute of Electronic Structure and Laser (FORTH-IESL), 70013 Heraklion, Greece

**Keywords:** biocompatibility, dental pulp stem cells, dental tissue engineering, odontogenic differentiation, scaffolds, tissue regeneration

## Abstract

This study aims to investigate the impact of kappa-carrageenan on dental pulp stem cells (DPSCs) behavior in terms of biocompatibility and odontogenic differentiation potential when it is utilized as a component for the production of 3D sponge-like scaffolds. For this purpose, we prepared three types of scaffolds by freeze-drying (i) kappa-carrageenan/chitosan/gelatin enriched with KCl (KCG-KCl) as a physical crosslinker for the sulfate groups of kappa-carrageenan, (ii) kappa-carrageenan/chitosan/gelatin (KCG) and (iii) chitosan/gelatin (CG) scaffolds as a control. The mechanical analysis illustrated a significantly higher elastic modulus of the cell-laden scaffolds compared to the cell-free ones after 14 and 28 days with values ranging from 25 to 40 kPa, showing an increase of 27–36%, with the KCG-KCl scaffolds indicating the highest and CG the lowest values. Cell viability data showed a significant increase from days 3 to 7 and up to day 14 for all scaffold compositions. Significantly increasing alkaline phosphatase (ALP) activity has been observed over time in all three scaffold compositions, while the KCG-KCl scaffolds indicated significantly higher calcium production after 21 and 28 days compared to the CG control. The gene expression analysis of the odontogenic markers DSPP, ALP and RunX2 revealed a two-fold higher upregulation of DSPP in KCG-KCl scaffolds at day 14 compared to the other two compositions. A significant increase of the RunX2 expression between days 7 and 14 was observed for all scaffolds, with a significantly higher increase of at least twelve-fold for the kappa-carrageenan containing scaffolds, which exhibited an earlier ALP gene expression compared to the CG. Our results demonstrate that the integration of kappa-carrageenan in scaffolds significantly enhanced the odontogenic potential of DPSCs and supports dentin-pulp regeneration.

## 1. Introduction

Progress in dental tissue engineering and regenerative dentistry has been tremendous over the past decades, with recent milestones on translational research having led to the development of innovative concepts in tissue engineering of hard and soft dental tissues, among which is the dentin-pulp complex [1]. Since the discovery of dental pulp stem cells (DPSCs), many biomaterials and signaling molecules have been investigated for their odontogenic response [2]. For example, in cases of direct and indirect pulp capping, various biocompatible platforms containing calcium and silicate-based materials have been utilized, aiming to reconstitute the exposed area and induce the odontogenic differentiation of the cells and the formation of a biomimetic mineralized barrier (tertiary dentinogenesis) [3].

Adult stem cells are considered to be a versatile model system and can be isolated from multiple organs of the human body. Their main advantage is their inert trait of self-renewal and their capability towards various differentiation lineages in the presence of appropriate stimulants [4,5]. DPSCs are a particular category of ectoderm-derived mesenchymal stem cells originating from migrating neural crest cells [6,7]. They reside in the pulp of permanent teeth and their main role is the production of odontoblasts and dentin. Moreover, the accessibility and the relatively easy process of isolation compared to other stem cell types, as well as their excellent behavior under in vitro conditions, make them a very promising cell type for experimentation in the regenerative medicine area [2,4,8]. For these reasons, DPSCs have been used in dental tissue engineering to evaluate their potential for dental reconstruction [9,10].

Scaffold-based therapies have been employed promisingly to achieve suitable matrices able to accommodate the regeneration of adjacent tissues [11]. Chitosan, a natural biopolymer that can be extracted from chitin, the main ingredient of the exoskeleton of different arthropod species such as shrimps, is one of the most commonly utilized biomaterials in tissue engineering [12]. Chitosan possesses alternating glucosamine and N-glucosamine groups to which it owes its excellent biocompatibility and antibacterial properties [12,13]. Based on its abundance and easy processability, chitosan has found applicability in various research fields, from the food industry [14] to the construction of tissue engineering scaffolds [15]. Additionally, it has been used as a promising scaffold for dentin and pulp tissue engineering [16]. One of the hurdles be overcome in the case of any potential regenerative medicine device is its ability to avoid an excessive inflammatory response, which can in turn lead to the rejection of the inserted transplant [17]. Gelatin is a natural biomaterial derived from hydrolyzed collagen comprising an alternating sequence of the arginine, glycine and aspartate (RGD) tripeptide motif, which promotes cell adhesion and a very low exhibition of immune responses [18]. Since gelatin is quite inexpensive while retaining most of the biological attributes of collagen, it is frequently employed in tissue engineering either in its native [19] or methacrylated form [20]. Due to their great biochemical response, chitosan and gelatin have been combined and studied, especially in the field of bone and dental tissue engineering [21,22,23]. For example, Georgopoulou et al. [15] reported on the preparation of chitosan/gelatin scaffolds, which were chemically crosslinked with a low concentration of either glutaraldehyde or genipin solution, to showcase the bone differentiation capacity of pre-osteoblasts and bone marrow derived mesenchymal stem cells (MSCs) in vitro and in vivo. Scaffolds depicted an excellent biocompatibility and upregulation of the examined osteogenic markers RUNX2, ALP and OSC up to day 14, while their insertion into a mouse femur illustrated the establishment of a well-spread extracellular matrix without adverse reactions. Capitalizing on the antibacterial properties of chitosan, Pereda et al. [24] reported on chitosan/gelatin edible films for long term food item preservation. Their results showed great antimicrobial activity, while the membranes were impenetrable to water, a crucial parameter for microbial growth inhibition.

Carrageenans are a family of various polysaccharides deriving from red seaweeds. Among them, kappa and iota-carrageenan have attracted great attention due to their gelling, thickening, stability and non-toxicity properties in food and commodity industries, but also as ingredients for regenerative medicine constructs due to their ability to promote cell growth, proliferation and differentiation [25,26,27]. The biological properties of kappa-carrageenan stem from its three hydroxyl groups per disaccharide repeating unit, which makes it highly hydrophilic, and its one negatively charged sulfate group, which enables chemical reactions [28]. Apart from the biocompatibility, tissue engineered constructs should possess sufficient mechanical stiffness similar to the tissue type targeted to regenerate to be successful [29]. In the presence of cations, especially monovalent ones such as potassium, kappa-carrageenan formation turns from a coil shape to a helicoidal structure, leading to increased gelling strength [30,31] and making it a prime candidate for tissue engineering applications [32].

Chitosan/gelatin scaffolds of various compositions have been previously examined for their odontogenic capacity, with promising in vitro and in vivo results [20,21]. In a previous study, we showed that the integration of kappa-carrageenan in chitosan/gelatin scaffolds positively influenced the mechanical properties and the osteogenic potential of pre-osteoblasts within the scaffolds [33]. In the context of dental applications, kappa-carrageenan has been described as a potent anti-human papillomavirus agent in cells and tissues of the oral cavity and as a hemostatic sponge when combined with gelatin, without any cytotoxicity in L929 fibroblasts [34,35]. However, there are no reports in the literature on tissue engineered carrageenan-based scaffolds for odontogenic differentiation. Therefore, in this study we aim to evaluate the impact of kappa-carrageenan in chitosan/gelatin scaffolds as a biomimetic microenvironment for dentin-pulp regeneration. In the present study, kappa-carrageenan/chitosan/gelatin (KCG) and kappa-carrageenan/chitosan/gelatin enriched with KCl (KCG-KCl) scaffolds have been produced, and the biological responses of DPSCs seeded onto them, including viability, proliferation, morphology and differentiation capacity towards the formation of mature odontoblasts, have been examined. Moreover, the mechanical strength of cell-loaded scaffolds was compared to that of cell-free scaffolds to deduce if the viscoelastic nature of DPSCs contributes to the reinforcement of the total robustness of the constructs, as an essential biomechanical attribute that controls cellular responses. To investigate the odontogenic capacity of the scaffolds, alkaline phosphatase (ALP) activity and calcium secretion were determined, and the gene expression levels of dental-related markers were evaluated by means of quantitative polymerase chain reaction (qPCR).

## 2. Results

### 2.1. Immunophenotypic Characterization of DPSC Cultures

**Figure 1:**
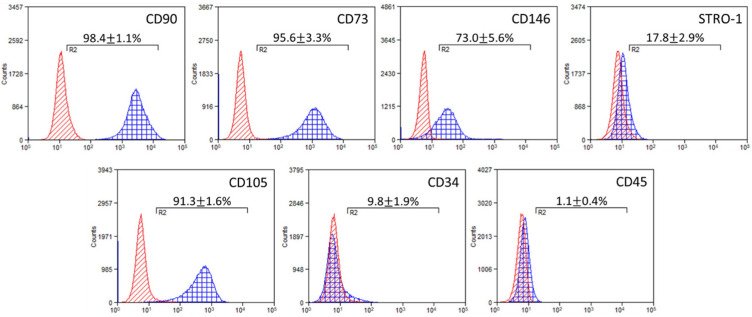
Representative histograms of immunophenotypic characterization by flow cytometry results on DPSCs surface markers: mesenchymal (CD90, CD73, CD146, STRO1), endothelial (CD105) and hematopoietic (CD34, CD45). The red area illustrates the expression of the control (unstained cells), and the blue area the marker of interest. The arithmetical values are the mean expression ± SD (*n* = 3).

The analysis of flow cytometry results revealed the high expression of MSC markers of the cell population, as illustrated in Figure 1. Specifically, CD90 was present at 98% and CD73 at 96% of the total cells compared to the control, and CD146 and STRO-1 at 73 and 18%, respectively. The endothelial cell marker CD105 was observed at 91%, while the hematopoietic markers CD34 and CD45 had minor expression (<10%). All expressed surface markers indicated a highly enriched DPSCs population.

### 2.2. DPSCs Contribution to the Mechanical Integrity of the Scaffolds

**Figure 2:**
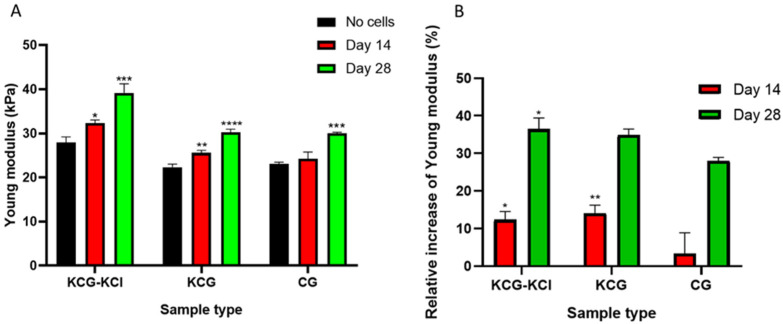
(**A**) Evaluation of the Young’s modulus at 5–20% strain and 1 mm/s velocity for the three scaffold compositions. (**B**) Relative expression of Young’s modulus increase of cell-loaded scaffolds between days 0 and 14 (red bars) and between days 0 and 28 (green bars) compared to cell-free scaffolds. Each bar represents the mean ± SD of *n* = 6 (* *p* < 0.05, ** *p* < 0.01, *** *p* < 0.001, **** *p* < 0.0001 compared to cell-free scaffolds (no cells)).

The Young’s modulus was measured at a strain of 5–20% and velocity of 1 mm/s (Figure 2) to monitor if the presence of cells affected the mechanical strength of the scaffolds. The measurements were performed under wet conditions, and the wet cell-free scaffolds were compared to the wet DPSCs laden scaffolds after 14 and 28 days in culture. Prior to the measurements, the excess of culture medium was removed from the scaffolds. Among the cell-free scaffolds, the KCG-KCl ones demonstrated the highest value, close to 28 kPa, while the KCG and control CG retained comparable values, approximately at 23 kPa. After 14 days in culture, all scaffolds showed a slight increase in the Young’s modulus, with the KCG-KCl indicating the highest value of 32 kPa, followed by the KCG at 25 kPa and the CG at 24 kPa. At day 28, all scaffold types exhibited the highest Young’s modulus values, with the KCG-KCl at 39 kPa and the other two at 30 kPa, demonstrating a 36% and 33% increase between day 0 and day 28, respectively. These findings strongly support the idea that the infiltration and proliferation of the cells inside the scaffolds led to mechanically enhanced constructs.

### 2.3. SEM Analysis of DPSCs Morphology and Adhesion

**Figure 3:**
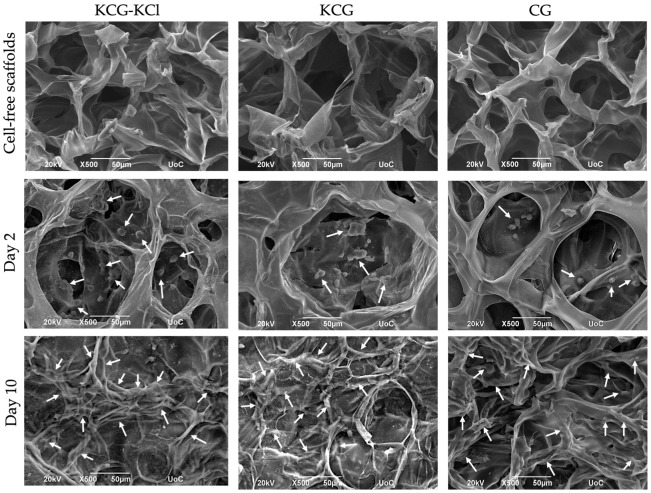
Representative SEM images from cell-free (upper panel) and cell-laden scaffolds after 2 days (middle panel) and 10 days (lower panel) in culture. KCG-KCl scaffolds (left column), KCG scaffolds (middle column) and CG control scaffolds (right column) are visualized. White arrows in the middle panel point to adhered cells and their nuclei on day 2, while in the lower panel the arrows point to dense elongated multicellular formations on day 10. Magnifications are 500× and scale bars represent 50 μm.

Scanning electron microscopy enables the illustration of the morphology of the scaffolds, the cell adhesion on their surface and the cell infiltration into the pores of the scaffolds. Figure 3 depicts representative SEM images of the three types of scaffolds, KCG-KCl, KCG and CG, either cell-free or loaded with DPSCs after 2 and 10 days of culture. Cell-free images are displayed in Figure 3 (upper panel) and exhibit the initially empty pores of the scaffolds that had been completely filled with cells by day 10. At day 2, adhered DPSCs could be detected within the pores, and the cell nuclei of healthy cells are also visible (white arrows point to the them). The morphology of attached cells on the scaffolds did not show any differences among the various compositions. At day 10, the pores of all three scaffold compositions were filled with elongated dense intercellular formations of DPSCs, signifying tissue growth.

### 2.4. DPSCs’ Growth and Proliferation within the Various Scaffold Types

**Figure 4:**
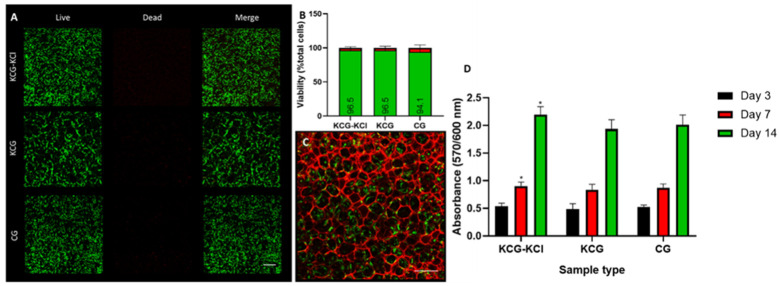
Cell viability assessment by CLSM live/dead fluorescent staining at 72 h and by the metabolic PrestoBlue^TM^ cell viability assay of DPSCs seeded on KCG-KCl, KCG and CG scaffolds at days 3, 7 and 14. (**A**) Representative CLSM (live and dead cells) images. (**B**) Mean percentage (%) of live to the total number of cells. Error bars indicate the standard deviation (*n* = 3). No statistically significant differences were observed among the groups. (**C**) CLSM image of the autofluorescent CG scaffold and the fluorescent cells, illustrating the homogenous distribution of the cells on the scaffold. Scale bars indicate 100 μm. (**D**) Cell viability assessment of DPSCs in the presence of the three scaffold types on days 3, 7 and 14. Each bar represents the mean ± SD of triplicates of two independent experiments (* *p* < 0.05, compared to the CG control scaffold at the corresponding time point).

All scaffold types demonstrated great biocompatible character, but no significant differences in their cytocompatibility levels could be detected (Figure 4). In detail, at day 3, all samples depicted similar absorbance values and, by day 7, almost a two-fold increase was evident for every composition compared to the previous time point, with the KCG-KCl one exceeding the rest by a small margin. At day 14, the same motif of growth was observed, with an almost three-fold increase in cell number compared to day 7 and with the KCG-KCl retaining a slightly higher value than the KCG and the CG control.

CLSM imaging confirmed that cells attached firmly and were evenly distributed on the scaffolds 72 h after seeding. Presentative CLSM photos of each scaffold composition are shown in Figure 4A. The green-appearing viable cells (as indicated by the enzymatic digestion of the fluorescent dye calcein AM) were predominant. The presence of red-appearing dead cells (stained by the ethidium bromide homodimer III) was limited in all groups. The percentage of live cells to the total cell number (live and dead) of each group is depicted in Figure 4Β. Games Howell’s post hoc test revealed no statistically significant differences among the tested groups (*p* > 0.05). DPSCs attached firmly in the CG scaffolds, as shown in Figure 3F.

### 2.5. Odontogenic Differentiation of DPSCs in the Presence of the Scaffolds

#### 2.5.1. Determination of the ALP Activity of DPSCs

**Figure 5:**
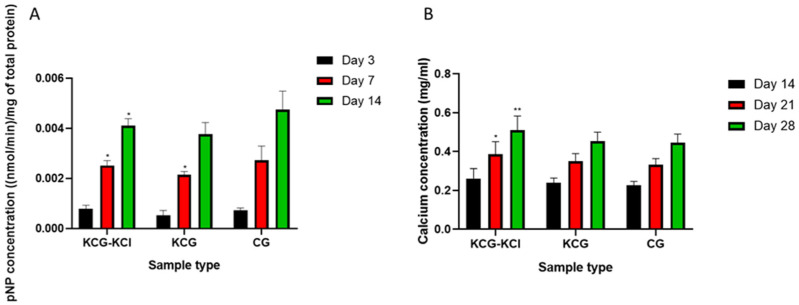
(**A**) Normalized alkaline phosphatase activity of DPSCs in the presence of the three scaffold types on days 3, 7 and 14. Each bar represents the mean ± SD of triplicates of two independent experiments (* *p* < 0.05 compared to the CG control scaffold at each time point). (**B**) Calcium concentration measured in the collected supernatants on days 14, 21 and 28 in culture from the three scaffold compositions. Each bar represents the mean ± SD of triplicates of two independent experiments (* *p* < 0.05, ** *p* < 0.01 compared to the CG control scaffold at each time point).

Since alkaline phosphatase activity is considered an early marker of odontogenesis, day 3, day 7 and day 14 were selected as the time points for its investigation (Figure 5A). At day 3, only a mild expression of the enzyme’s activity was evident, with all scaffold types having identical values. Between day 3 and day 7, a steep increase was observable for all compositions of approximately quadruple magnitude, with the KCG-KCl and CG scaffolds exhibiting slightly higher values than the KCG ones. Finally, at day 14, all scaffolds showed a two-fold increase, with no profound variations between them.

#### 2.5.2. Evaluation of the Secreted Calcium in Supernatants

The concentration of secreted calcium secreted by the DPSCs was quantified on days 14, 21 and 28 (Figure 5B). All samples showed not only similar values with regard to calcium concentration, but also an identical trend in the way it increased. Specifically, at day 14, the KCG-KCl scaffolds slightly surpassed the other two compositions, with the same motif being repeated for the other two time points as well. KCG and CG samples retained similar values throughout the 28 days period, at all different time points. The increase of calcium concentration was linear between the subsequent time points, with an almost 50% increase between the previous and the next time point.

#### 2.5.3. Real-Time PCR Odontogenic Markers

**Figure 6:**
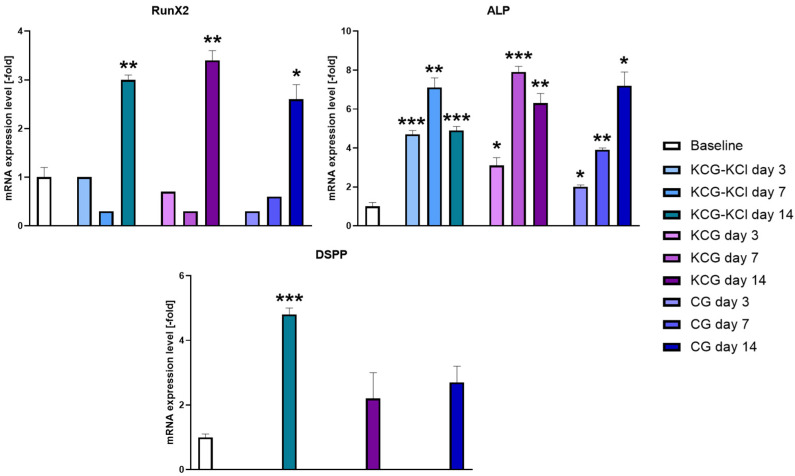
Odontogenic differentiation of DPSCs after 3, 7 and 14 days in contact with the tested scaffolds. The mRNA expressions of *RunX2*, *ALP* and *DSPP* are expressed as fold change compared to the two housekeeping genes (*B2M* and *SDHA*). DPSCs that were scaffold-free acted as the baseline. Asterisks indicate the statistically significant differences compared to the negative control (* *p* ≤ 0.05, ** *p* ≤ 0.01, *** *p* ≤ 0.001) (*n* = 3).

The gene expression of odontogenic markers was upregulated at different levels in all three tested scaffolds, as assessed by real-time PCR at 3, 7 and 14 days. The KCG-KCl scaffold showed the highest odontogenic effect on DPSCs. Gene expressions normalized to the two HKGs (*SDHA* and *B2M*) are displayed as fold change and illustrated in Figure 6. More specifically, the expression of *DSPP*, which is a late marker of odontogenic differentiation, was not expressed until day 14 in any of three examined DPSC-seeded scaffolds. CG and KCG compositions revealed an approximately two-fold upregulation, compared to baseline control, while a statistically higher (five-fold) value was identified at day 14 of treatment inside the KCG-KCl scaffolds (*p* < 0.001). Similarly, the expression of the *RunX2* gene was upregulated only at day 14, for all groups, with an approximately three-fold increase, while the *ALP* marker expression levels of KCG-KCl and KCG scaffolds depicted a seven-fold and eight-fold increase at day 7, respectively, followed by a decline at day 14. Conversely, CG scaffolds showed a continuous upregulation profile, up to day 14, which peaked at a seven-fold increase at the last time point. 

## 3. Discussion

Oral-cavity-related diseases belong to the most prevalent categories of health problems, encompassing a wide range of pathophysiologies from the more typical tooth and periodontal damage to life threatening cancers [34]. DPSCs are stem cells that can be isolated from the dental pulp of permanent teeth and are capable of being guided towards different lineage pathways, making them an ideal cell line to work with for the investigation of regenerative model systems [4]. One of their most popular applications is related to their directed differentiation towards odontoblast-like cells to enable the recuperation of dental tissue [23]. Research in tissue regeneration strives to propel current medicinal standards further. To this end, researchers have investigated the development of novel treatment methods that can accommodate the restoration of tissue defects caused by exogenous trauma or health issues resulting from genetic abnormalities [35]. Dentin and pulp tissue engineering would significantly benefit dental clinical practice, and therefore recent studies have focused on the discovery of new bioactive materials to facilitate this purpose. Indirect and direct pulp capping materials have been utilized throughout the years to accommodate induced odontogenesis at the site of pulp exposure or proximity [2].

Biomaterials have been developed over the last decades as a source for the construction of cell friendly platforms that interact with the human body and enable gradual regeneration in a desired, controllable way. They can be broadly classified into two categories based on their origin and the method of their production, natural and synthetic, and have been used in various combinations to create tissue engineering scaffolds that enhance the growth of specific tissue types. Natural biomaterials are similar to structural biomolecules that compose native tissue, and thus their biological effect is usually more potent than that of the synthetic ones [36]. However, one of the main downsides of natural biopolymers is their low mechanical strength, making it pivotal to either crosslink or mix them with other, firmer compounds. Chitosan and gelatin have been extensively studied in tissue engineering applications, due to their superb biological response towards different cell lines [15,23]. Chitosan scaffolds, in combination with various bioactive substances, have been investigated for their dentin and pulp tissues regeneration ability with advantageous results [11]. Kappa-carrageenan, a natural biocompatible polysaccharide, possesses a negatively charged sulfate group which provides the capability to form firm gels at room temperature in the presence of a cation such as sodium or potassium [30]. Moreover, the spare sulfate groups that do not interact with these cations are free to form ionic binding with other positively charged groups. This property can be exploited when kappa-carrageenan and chitosan are combined to form polyelectrolyte complexes that stem from the ionic interactions occurring between the positively charged amino groups of chitosan and the negatively charged sulfate groups of kappa-carrageenan, leading to an amplification of the scaffolds’ mechanical strength [37]. In this study, we fabricated two types of kappa-carrageenan-containing scaffolds with and without the addition of potassium chloride based on their capacity to promote the osteogenic responses of pre-osteoblastic cells [33]. We attempted to illustrate their contribution to induce the odontogenic differentiation of DPSCs towards odontoblast-like cells. Chitosan/gelatin scaffolds served as the control group.

Under wet conditions, all three types of scaffolds behaved as sponge-like materials, able to absorb large quantities of water, with Young’s modulus values ranging between 22.5 and 28.5 kPa. The KCG-KCl indicated the highest and the KCG and CG had almost identical values, underlying the impact of potassium chloride in the overall mechanical robustness of the scaffolds. Such structures of spongy nature have been proven to allow and promote bone differentiation [38]. Although the produced scaffolds provided an excellent biocompatible 3D environment for in vitro biological evaluation, their elastic modulus values were mechanically inferior compared to the native dental pulp tissue [39], which may limit their use as substitutes. Regarding degradable materials, when cells are seeded onto scaffolds, two antagonistic procedures constantly take place: the degradation of the scaffold, and the de novo synthesis of the extracellular matrix by the cells. The degradation rate of the three scaffolds compositions, as well as the role of kappa-carrageenan and the crosslinker KCl in reducing the degradation rate of the chitosan/gelatin scaffolds, has been thoroughly investigated and reported on previously [33]. Different research groups have shed light on the viscoelastic attributes of various cell lines, which derive from the intricate complexes that their different structural biomolecules formulate [40,41]. To evaluate whether the cells contribute to the stiffness of the fabricated scaffolds, measurements of the Young’s modulus were conducted after 14 and 28 days of cell culture. Between days 0 and 14, a slight increase of approximately 12% was observed for the two kappa-carrageenan containing scaffolds, with the KCG-KCl exceeding KCG and the CG scaffolds showing a 4% increase. However, at day 28, a significant rise to 39 kPa was detected for the KCG-KCl, followed by the KCG and CG scaffolds at 30 kPa. All scaffolds showed increased elastic modulus values by a range of 27–36% compared to cell-free scaffolds. These significant differences reinforced our initial hypothesis: that the presence of DPSCs have a positive effect on the elastic modulus of the cell laden scaffolds. These results are in alignment with other research works reporting that cells can show elastic modulus values of several kPa [42,43].

All scaffolds showed excellent biocompatibility, with the living cell numbers gradually climbing towards higher values over time. The differences in the absorbance values were almost negligible, with the KCG-KCl scaffolds surpassing the other types at all time points. Scaffold porosity is a crucial parameter for a tissue engineering construct, as it can directly affect cell migration and infiltration after the saturation of the surface [44]. In a previous study, we showed that these scaffold types displayed a porosity of at least 80% [33]. This architecture allowed for a deep infiltration of the pores by the DPSCs after surface saturation, resulting in a cell number increase up to day 14 [45]. SEM images at day 2 illustrated that the cells had adhered to the material surface, and by day 10 dense elongated intercellular formations covering the pores of the scaffolds were visible. These findings indicated tissue growth formation, and they were confirmed by the live/dead staining results, revealing excellent biocompatibility. The living cells were distributed within the scaffolds, as illustrated by the CLSM images. These findings are in accordance with reports from the literature on chitosan scaffolds evaluated in direct contact with DPSCs [22,46].

Alkaline phosphatase activity is a crucial marker of odontogenesis since the enzyme is able to cleave phosphate groups from different molecules and make them available for the later stage of mineralization. As such, the enzyme activity is expected to have high expression during the early odontogenesis phase, and to decline afterwards [47]. All three scaffold types showed almost a two-fold increase of the ALP activity between days 3 and 7, with the same motif being evident for the period between days 7 and 14. The CG control had the highest values at all time points; however, this was not significant compared to the other two compositions. Calcium secretion is representative of the final stage of odontogenesis and is essential for the formation of hydroxyapatite, the main inorganic component of dental tissue. It is considered a middle-to-late marker of odontogenesis [48]; thus, it was monitored at days 14, 21 and 28. At day 14, all scaffolds depicted similar calcium secretion levels. Between days 14 and 21, a rise in calcium production was detected for all scaffold compositions, and similarly up to day 28. The KCG-KCl scaffolds indicated significantly increased calcium production on days 21 and 28 compared to the CG control. The results from both odontogenesis assays coincided with the findings from our previous work, investigating the pre-osteoblasts maturation, as well as with other studies focusing on the differentiation capability of kappa-carrageenan-containing scaffolds [33,47,49].

The odontogenic effect of all three scaffold compositions was investigated by means of qPCR (Figure 6). The KCG-KCl scaffolds showed the optimal odontogenesis induction effect, as indicated by the *DSPP* expression. *DSPP* belongs to the family of Small Integrin-Binding Ligand N-linked Glycoproteins (SIBLINGs), and is an odontogenic specific marker [50]. This protein participates in the mineralization of the dentin matrix and has been found to be involved in bone mineralization as well, but on a lesser scale [51]. In our study, *DSPP* expression was upregulated at day 14 in all three scaffold groups, with only the KCG-KCl scaffolds displaying a statistically significant increase. Even though *RunX2* and *ALP* are not specific markers for odontogenic differentiation, their effect on the mineralization of the dentin matrix is essential [52]. *RunX2* expression increased at day 14 in all three investigated scaffolds, while the expression of *ALP* was statistically higher than the baseline at all timepoints. Interestingly, the ALP expression was more upregulated at days 3 and 7 in the kappa-carrageenan-containing scaffolds, compared to the CG control, with a decline on day 14, while the CG scaffolds retained their highest values at this time point, implying that the odontogenic process started earlier in the carrageenan containing scaffolds. It has been shown that when dexamethasone, a general induction factor, was applied on DPSCs, the expression of *RunX2* was not upregulated [53], while in this study all tested scaffold compositions caused increased *RunX2* expression. The upregulation of the expression of the transcription factor *RunX2* is essential for the odontogenic differentiation of DPSCs to odontoblast-like cells [54]. It has also been shown that bioactive pulp-capping materials that are commonly used in dental clinical praxis, such as silicate cements and mineral trioxide aggregate (MTA), do not affect the *ALP* gene expression, while CG, KCG and KCG-KCl significantly upregulated this gene [55]. In previous works of our group revolving around the odontogenic potential of chitosan/gelatin-based scaffolds, we have shown the upregulation of these markers in a similar 3D environment [23,56]. The abovementioned findings strongly indicate the odontogenic stimuli of the CG, KCG and KCG-KCl scaffolds as early as day 3. The sponge-like architecture of the produced scaffolds favors the physical absorption of high amounts of water, thus rendering them excellent candidates for enhanced functional platforms for the absorption and subsequent release of drugs, growth factors and various biomolecules, as well as bioactive inorganic compounds that are dissolvable in biological environments.

## 4. Materials and Methods

### 4.1. Materials

Chitosan (low molecular weight), gelatin from bovine skin, kappa-carrageenan, potassium chloride, magnesium chloride, glutaraldehyde, Triton X-100, p-nitro-phenyl-phosphate (pNPP), L-ascorbic acid 2-phosphate, β-glycerophosphate and dexamethasone were purchased from Sigma-Aldrich (St. Louis, MO, USA). The PrestoBlue^TM^ reagent for cell viability and proliferation was purchased from Invitrogen Life Technologies (Carlsbad, CA, USA). Trypsin/EDTA (0.25%), phosphate-buffered saline (PBS), amphotericin-B (fungizone), L-glutamine and penicillin/streptomycin (P/S) were all purchased from Gibco ThermoFisher Scientific (Waltham, MA, USA). Minimum essential Eagle’s medium (alpha-MEM) and fetal bovine serum (FBS) were purchased from PAN-Biotech (Aidenbach, Germany). O-cresol phthalein complexone reagent kit (CPC) was purchased from Biolabo (Les Hautes Rives Maizy, France). Bradford reagent was purchased from AppliChem GmbH (Darmstadt, Germany). Collagenase I was purchased from Abiel Biotech (Palermo, Italy), and dispase II from Roche (Basel, Switzerland). The viability assay kit for live/dead staining (calcein AM and EthD-III) was purchased from Biotium (Fremont, CA, USA), the NucleoSpin RNA isolation kit from Macherey-Nagel (Duren, Germany), the cDNA Synthesis kit PrimeScript first-strand from Takara (Shiga, Japan), and the SYBR PCR Master Mix from Applied Biosystems (Foster City, CA, USA). The fluorochrome-conjugated antibodies for cell surface marker characterization were purchased from BioLegend (Fell, Germany).

### 4.2. Scaffolds Preparation

**Scheme 1:**
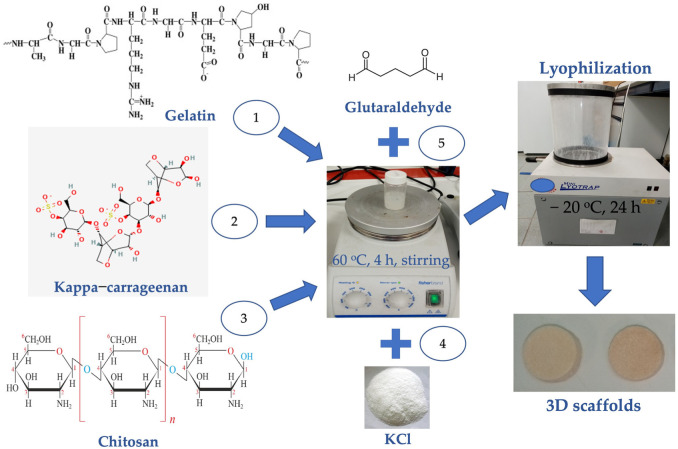
Graphical representation of the scaffold production. The numbers 1–5 in the scheme show the sequential addition of each compound (1 of gelatin, 2 of kappa carrageenan, 3 of chitosan, 4 of KCl and 5 of glutaraldehyde).

Three types of scaffolds were produced following a protocol based on our previous work [33]: (i) kappa-carrageenan/chitosan/gelatin (KCG), (ii) kappa-carrageenan/chitosan/gelatin enriched with potassium chloride (KCG-KCl) and (iii) chitosan/gelatin (CG) acting as a control. For the fabrication of the scaffolds, an established protocol was followed based on our previous work [33]. Briefly, for the KCG scaffolds a 2.5% *w*/*v* chitosan in 1% *v*/*v* acetic acid solution was allowed to mix at 50 °C for 1 h, while another 5% *w*/*v* gelatin solution was prepared in deionized water for 20 min at 50 °C. The two solutions were mixed in a volume ratio of 2:1 chitosan/gelatin, and afterwards the final solution was mixed with a 1% *w*/*v* kappa-carrageenan water solution. The KCG-KCl mixtures were prepared in exactly the same way, but with the addition of 0.25 M KCl. Subsequently, the resulting solutions were left under mild stirring for 4 h at 60 °C to homogenize completely. From each of them, 10 mL were transferred into a 15 mL tube and, after the addition of 50 μL of 0.25% *v*/*v* glutaraldehyde, 800 μL of the blend was cast onto the wells of a 24 well-plate and freeze-dried for 24 h to create porous 3D scaffolds. Prior to cell seeding, the scaffolds were UV treated at 262 nm to disinfect them for 30 min. Table 1 presents the scaffold designation and their chemical composition, while Scheme 1 summarizes the production process.

### 4.3. Establishment and Culture of DPSCs

The protocol of this study received approval from the Institutional Ethics Board Committee (number 46/20-3-2019). Three patients donated their healthy third molars, which were extracted for orthodontic/preventive reasons. Dental pulp stem cell pulled cultures were established using the enzymatic dissociation method, as described previously [57]. Briefly, immediately after tooth extraction teeth were cleaned from soft remnant tissues and disinfected with iodine. The pulp chamber was exposed after a cut was performed through the cemento-enamel junction of each tooth using a high-speed handpiece instrument. The pulp tissue was transported in serum-free cell culture medium to sterile conditions. The pulp was then mechanically minced and enzymatically dissociated for 45 min at 37 °C in a buffer solution of 3 mg/mL collagenase I and 4 mg/mL dispase II.

DPSCs were cultured in complete culture medium (CCM), consisting of a minimum essential medium supplemented with 15% fetal bovine serum (FBS), 0.1 mΜ L-ascorbic acid and a triple antibiotic solution of 100 U/mL penicillin, 0.1 mg/mL streptomycin and 0.25 μg/mL amphotericin B. Cells’ incubation conditions were 37 °C, 5% CO_2_ and 100% relative humidity (RH) (Heal Force, Shanghai, China) until 70–80% confluency was reached. Cell detachment was performed by 0.25% Trypsin/1 mM EDTA, and passages of 2–6 were used for all experiments. For the differentiation experiments, the medium was enriched with 50 μg/mL l-ascorbic acid, 10 mM β-glycerophosphate and 10 nM dexamethasone.

### 4.4. Immunophenotypic Characterization of DPSCs

DPSCs were characterized for specific surface markers by flow cytometry. Cells were suspended, washed with flow cytometry staining buffer (FCSB) containing 1% BSA and 0.1% NaN3 in PBS, and stained for the relevant markers. The mesenchymal (STRO1, CD146, CD90, CD73), endothelial (CD105), embryonic (SSEA4) and hematopoietic (CD34, CD45) surface markers on single-cell suspensions were labelled with the following fluorochrome-conjugated antibodies: CD90-FITC, CD34-APC, CD45-PE, SSEA4-FITC, STRO1-APC, CD146-PE, CD105-APC and CD73-PE. Data were acquired from 50.000 events per sample using a Guava EasyCyte 8HT Benchtop flow cytometer (Merck Millipore, Darmstadt, Germany) and analyzed with the Summit 5.1 software (Summit Software Technologies, Fort Wayne, IN, USA) (*n* = 3).

### 4.5. Cell Viability Assessment

#### 4.5.1. Cell Viability Assessment with the PrestoBlue^TM^ Metabolic Assay

A 10 μL cell suspension containing 8 × 10^4^ cells was seeded onto each scaffold and 400 μL of fresh medium was added to a final volume of 400 μL for each well of a 24 well-plate. The cell viability of the scaffolds was investigated by employing PrestoBlue^TM^ assay, whose base ingredient is resazurin, a reductive agent that changes color from blue to purple according to cell metabolism levels. Briefly, based on an established protocol [33], at three different time points, days 3, 7 and 14, the old medium for each scaffold was replaced with a mixture of 40 μL of PrestoBlue^TM^ and 360 μL of fresh medium and the 24 well-plate was incubated for 1 h at 37 °C. Subsequently, 100 μL of this mixture was transferred to a 96 well-plate and were photometrically measured with a spectrophotometer at 570 and 600 nm (Synergy HTX Multi-Mode Micro-plate Reader, BioTek, Bad Friedrichshall, Germany). All samples were analyzed in triplicates of two independent experiments (*n* = 6).

#### 4.5.2. Cell Viability Assessment by Live/Dead Staining

Scaffolds containing 24-well plates were seeded with 10^5^ cells/well in 1 mL CCM/well. The cell-seeded scaffolds were cultured further for 3 days, and then cell viability was assessed by live/dead double-staining using 2 mM calcein AM ((λex/λem(max): 494/517 nm)/4 mM ethidium homodimer III ((λex/λem(max): 532/625 nm) for up to 45 min. Calcein AM was cleaved by esterases of live cells to produce the green fluorescent dye calcein, while EthD-III stained the nucleus of dead cells with bright red fluorescence. The seeded HTFD scaffolds were observed by confocal laser scanning microscopy (CLSM) (Leica Microsystems, Wetzlar, Germany). Approximately 35 steps of 10 μm-step size z-stacked images were generated. Two images were taken per scaffold. Cells seeded on a glass surface acted as a negative control. The numbers of live and dead cells were measured with the ImageJ software (version 1.8.0, NIH, Bethesda, MD, USA). The % percentage of live cells to the total number of cells was calculated (*n* = 3 per condition). When the gains of the infra-red laser were increased, the structure of the scaffold was observed and cell morphology, attachment and distribution were observed.

### 4.6. Mechanical Properties of the DPSCs Seeded Scaffolds

The various scaffold compositions were examined mechanically by determining the differences of the Young’s modulus values between cell seeded and cell-free scaffolds. The elastic modulus value of the cells can play an important role to the reinforcement of the mechanical integrity of the final construct, and thus it is an important parameter to take into account [58]. Compression testing was conducted by means of a compression and tensile strength test unit (UniVert, CellScale, Waterloo, ON, Canada) with a maximum cell load of 50 N. The measurements were conducted under wet conditions (*n* = 6). The control cell-free scaffolds were compared to DSPCs laden scaffolds after 14 and 28 days of culture. The Young’s modulus was calculated at a strain of 5–20% and velocity of 1 mm/s using the following formula:

$$Young\;modulus:\frac{F\times L}{A\times \Delta l}$$

where *F* is the perpendicular force applied to the surface of the scaffolds, *A* is the surface, Δ*l* is the height strain of the scaffolds after the force application and *L* is the initial height.

### 4.7. Scanning Electron Microscopy (SEM)

The morphology of both the cell-free and cell laden scaffolds was investigated by means of scanning electron microscopy (JEOL JSM-6390 LV) at days 2 and 10. The cell-laden scaffolds were rinsed twice with PBS and fixed using a PFA 4% solution. The scaffolds were then dehydrated by employing increasing ethanol concentrations, from 30 to 100% pure ethanol. For the cell-free scaffolds, the same procedure was followed but without fixation. At the last stage, the scaffolds were dried in a critical point drier (Baltec CPD 030), sputter-coated with an 80 nm thick layer of gold (Baltec SCD 050) and observed under a scanning electron microscope at an accelerating voltage of 20 kV (JEOL JSM-6390 LV).

### 4.8. Odontogenic Differentiation

#### 4.8.1. Alkaline Phosphatase Activity

The ALP activity is indicative of the early stages of odontogenesis. For these experiments, 1 × 10^5^ DPSCs were seeded onto the different scaffold compositions and the enzyme activity was evaluated at days 3, 7 and 14. The scaffolds were rinsed with PBS multiple times, and then incubated in a solution comprising 0.1% Triton X-100 and 50 mM Tris-HCl, at pH 10.5, so that cell lysis can take place. After three freezing/thawing cycles between room temperature and −20 °C, a 100 μL suspension from each well was mixed with 100 μL of a 2 mg/mL p-nitro-phenyl-phosphate (pNPP) solution diluted in a buffer containing 50 mM Tris-HCl and 2 mM MgCl_2_. The resulting mixtures were allowed to incubate at 37 °C, and then 100 μL from each well was transferred to a 96-well plate and photometrically measured at 405 nm in a spectrophotometer (Synergy HTX Multi-Mode Micro-plate Reader, BioTek, Bad Friedrichshall, Germany) [59]. The enzymatic activity was calculated using the equation [units = nmol p-nitrophenol/min] and normalized to total cellular protein in lysates determined using the Bradford protein concentration assay (AppliChem GmbH, Darmstadt, Germany). All samples were analyzed in triplicates of two independent experiments (*n* = 6).

#### 4.8.2. Calcium Secretion Levels Determination

The production of calcium is connected with the formation of hydroxyapatite, one of the basic and most crucial components of bone and dental tissue. The O-cresol phthalein complexone (CPC) method was used to determine the calcium concentration in the supernatants that were collected every three days of cell culture, up to day 28. Briefly, 10 μL of culture medium from each sample was mixed with 100 μL of calcium buffer and 100 μL of calcium dye, as previously described [33], and transferred to a 96-well plate for photometrical measurement at 550 nm in a spectrophotometer (Synergy HTX Multi-Mode Micro-plate Reader, BioTek, Bad Friedrichshall, Germany) [60]. All samples were analyzed in triplicates of two independent experiments (*n* = 6).

#### 4.8.3. Gene Expression of DPSCs

Scaffolds were placed in 24-well plates and were seeded with 6 × 10^5^ cells/well, in 1 mL CCM/well for 24 h. The effect of odontogenic differentiation was evaluated at 3, 7 and 14 days by quantitative real-time polymerase chain reaction (qPCR), as described previously [23]. A group consisting of cells alone, without the presence of scaffolds, acted as a negative control (baseline expression group). Cells were lysed and the RNA was isolated using the NucleoSpin RNA isolation kit. The cDNA was then synthesized using the PrimeScript first-strand cDNA Synthesis Kit. Finally, qPCR was performed in a thermal cycler (Step One Plus, Applied Biosystems, Waltham, MA, USA) using the SYBR PCR Master Mix. Two incubation steps at 50 °C for 2 min and at 95 °C for 2 min were followed by 40 cycles of PCR, comprising denaturation for 15 s at 95 °C and annealing/extension for 1 min at 60 °C. The relevant primers (Table 1) were designed with the Primer-Blast software (NCBI, NIH, Bethesda, MD, USA). Dehydrogenase complex, subunit A, flavoprotein (*SDHA*) and beta-2-microglobulin (*B2M*) were used as house-keeping genes (HKGs). The genes of interest (GOI) were the following: dentin sialophosphoprotein (*DSPP*), alkaline phosphatase (*ALP*) and the transcription factor Runt-Related Transcription Factor-2 (*RUNX2*) (Table 2). The results were adjusted by amplification efficiency with the LinRegPCR software (Amsterdam, The Netherlands) and normalized to the HKGs. The quality of amplification and specificity were checked by the amplification plot and the standard melting curves. The expression of GOI was normalized to the HKGs, displayed as a fold expression and statistically analyzed (*n* = 3).

### 4.9. Statistical Analysis

Statistical analysis for the assessment of cell viability, ALP activity and calcium production was performed using the one-way ANOVA Dunnett’s multi-comparison test in GraphPad Prism version 8 software (GraphPad Software, San Diego, CA, USA), comparing each kappa-carrageenan-containing scaffold with the CG control at each experimental time point. A *p*-value < 0.05 was considered significant. The results of live/dead staining were analyzed with two-way ANOVA followed by Tukey’s post hoc test. PCR results for the relevant gene expression were analyzed by Welch’s ANOVA (Kolmogorov–Smirnov *p* > 0.05), followed by Games Howell’s post hoc test. Data were expressed as means ± standard deviation (SD).

## 5. Conclusions

This study investigated the role of kappa-carrageenan in odontogenesis promoting when incorporated in a chitosan/gelatin biomimetic matrix, which is widely regarded as one of the most favorable scaffolds for mineralized tissue engineering. Both kappa-carrageenan containing scaffolds exhibited great biocompatible and odontogenic differentiation capabilities. Real-time PCR analysis revealed an earlier expression of the ALP marker for the two scaffolds, compared to the control, while KCG-KCl samples had the highest upregulation of the odontogenesis specific DSPP marker at day 14. Moreover, all scaffold compositions showed an increase in Young’s modulus values with progressing culture time, validating our initial hypothesis of the cell contribution to the reinforcement of the scaffolds’ total mechanical robustness. These results underline that kappa-carrageenan scaffolds offer an excellent 3D environment for DPSCs, with a remarkable potential for dentin-pulp reconstitution. However, more specific biological markers could be evaluated in the future to gain a better insight into the regeneration capacity of the complex dentin-pulp tissue using kappa-carrageenan scaffolds. In addition, the use of carrageenans as dental pulp implants is still weighed down by their relatively low mechanical strength, giving room for further future experimentation.

## Figures and Tables

**Table 1:** Acronyms of the various scaffold compositions.

Type	Scaffold Acronym	Composition
(i)	KCG-KCl	1% *w*/*v* kappa-carrageenan, 1.67% *w*/*v* chitosan, 1.67% *w*/*v* gelatin, 0.25 M KCl
(ii)	KCG	1% *w*/*v* kappa-carrageenan, 1.67% *w*/*v* chitosan, 1.67% *w*/*v* gelatin
(iii)	CG	1.67% *w*/*v* chitosan, 1.67% *w*/*v* gelatin

**Table 2:** Primers design and amplicon size for the genes of interest and housekeeping genes used in the qPCR analysis.

Gene Symbol	Forward (5′–3′)	Reverse (5′–3′)	Amplicon Size (bp)
*DSPP*	GCTGGCCTGGATAATTCCGA	CTCCTGGCCCTTGCTGTTAT	135
*ALP*	CCGTGGCAACTCTATCTTTGG	CAGGCCCATTGCCATACAG	89
*RUNX2*	CCACCGAGACCAACAGAGTC	TCACTGTGCTGAAGAGGCTG	118
*B2M*	TGTCTTTCAGCAAGGACTGGT	ACATGTCTCGATCCCACTTAAC	138
*SDHA*	GCATGCCAGGGAAGACTACA	GCCAACGTCCACATAGGACA	127

## Data Availability

Data can be made available upon request.
